# Single Molecule *In Vivo* Analysis of Toll-Like Receptor 9 and CpG DNA Interaction

**DOI:** 10.1371/journal.pone.0017991

**Published:** 2011-04-04

**Authors:** Jiji Chen, Suman Nag, Pierre-Alexandre Vidi, Joseph Irudayaraj

**Affiliations:** 1 Bindley Bioscience Center and Birck Nanotechnology Center, Department of Agricultural and Biological Engineering, Purdue University, West Lafayette, Indiana, United States of America; 2 Department of Chemical Sciences, Tata Institute of Fundamental Research, Mumbai, India; 3 Department of Basic Medical Sciences, Purdue University, West Lafayette, Indiana, United States of America; New England Biolabs, Inc., United States of America

## Abstract

Toll-like receptor 9 (TLR9) activates the innate immune system in response to oligonucleotides rich in CpG whereas DNA lacking CpG could inhibit its activation. However, the mechanism of how TLR9 interacts with nucleic acid and becomes activated in live cells is not well understood. Here, we report on the successful implementation of single molecule tools, constituting fluorescence correlation/cross-correlation spectroscopy (FCS and FCCS) and photon count histogram (PCH) with fluorescence lifetime imaging (FLIM) to study the interaction of TLR9-GFP with Cy5 labeled oligonucleotide containing CpG or lacking CpG in live HEK 293 cells. Our findings show that i) TLR9 predominantly forms homodimers (80%) before binding to a ligand and further addition of CpG or non CpG DNA does not necessarily increase the proportion of TLR9 dimers, ii) CpG DNA has a lower dissociation constant (62 nM±9 nM) compared to non CpG DNA (153 nM±26 nM) upon binding to TLR9, suggesting that a motif specific binding affinity of TLR9 could be an important factor in instituting a conformational change-dependant activation, and iii) both CpG and non CpG DNA binds to TLR9 with a 1∶2 stoichiometry *in vivo*. Collectively, through our findings we establish an *in vivo* model of TLR9 binding and activation by CpG DNA using single molecule fluorescence techniques for single cell studies.

## Introduction

Toll-like receptors (TLRs), one of the pattern-recognition receptors (PRRs), are the key sensors of microbial infection in mammals [1], [2]. Human TLRs have been identified with different sub-cellular localizations and have been found to recognize a wide array of molecules comprising of lipopolysaccharides and nucleic acids [3], [4], [5], [6]. TLR9 is thought to be able to activate the innate immune system by detecting unmethylated CpG dinucleotides, which are common in the genomes of most bacterial and DNA viruses, but which are suppressed and methylated in vertebrate genomes [7]. Ligand binding to TLR9 results in the recruitment of adaptor proteins, MyD88, to ultimately lead to the activation of NF-κB, a key regulator of many inflammatory response pathways [8]. MyD88 has a C-terminal Toll/IL-1R (TIR) containing portion that associates with the TLR-TIR domain and an intermediate domain (ID) that is crucial in TLR signaling since it interacts with IL-1R associated kinases (IRAKs) [9].

Although the prevailing paradigm attributes the activation of TLR9 to the recognition of CpG containing DNA, reports present different explanation on the ability of TLR9 to discriminate nucleic acids based on *in vitro* studies. Rutz *et al.* found that TLR9 interacts with nucleic acids in a sequence-specific manner [10] while others report that TLR9 binds to nucleic acids in a sequence-independent manner [11], [12], [13]. It was further demonstrated that binding to CpG DNA could induce conformational changes and subsequently reduce the diameter of the extracellular domain [14]. However, all of these investigations relating to the interaction of TLR9 with nucleic acids are based upon *in vitro* assay, no report has examined such interactions in live cells. Tools to elucidate TLR9 activation or inactivation *in vivo* have the potential to broadly impact TLR9 biology and assist in the development of more effective therapeutics.

Since most of the past attempts use traditional biochemical techniques, which generally require the disruption of natural cellular compartments, interrogation of the intracellular kinetics was not possible. Single molecule fluorescence techniques such as fluorescence correlation/cross-correlation spectroscopy (FCS/FCCS) and photon counting histogram (PCH) provides a high spatial and temporal resolution for direct quantitative investigation of molecular dynamics in live cells. FCS allows for the non-invasive *in vivo* monitoring of the diffusion time (τ_D_) and the absolute concentration of fluorescing probes diffusing through the confocal volume (< 1fL) [15], [16], [17]. While FCS examines the autocorrelation of fluorescence from a single species, FCCS examines the fluorescence signal from two species simultaneously through cross-correlation to provide quantitative information of the number of molecules and their interaction [18], [19], [20]. PCH could be used to evaluate molecular brightness (expressed as counts per second per molecule, cpsm) to estimate the stoichiometry (monomers, dimers, trimers, etc) [21], [22] of the diffusers from fluorescence fluctuation data.

In this study, we combine FCS/FCCS, PCH and fluorescence lifetime imaging (FLIM) to investigate the interaction of DNA containing CpG or lacking CpG in HEK 293 cells expressing TLR9-GFP. Through quantitative PCH analysis, we find that TLR9 predominantly forms homodimers before binding to the nucleic acids and the percentage of dimers does not change upon interaction with either CpG or non CpG DNA. Through FCS we reveal that CpG-TLR9 complex has different diffusion dynamics compared to non CpG-TLR9. With FCCS we further observe that both CpG and non CpG DNA binds to TLR9 in a 1∶2 stoichiometry *in vivo* but with different affinity. While the integrated approach used in this study provides a noninvasive and quantitative means to understand TLR9 and nucleic acid interactions *in vivo*, the demonstrated single molecule analysis tools in single cells can be extended for studies with other protein-ligand interaction in live cells.

## Results

### PCH analysis reveals TLR9 forms homodimers in live cells

The formation of higher order oligomers possibly representing a conserved mechanism of activation has been demonstrated as a signaling prerequisite for a number of TLRs [23], [24]. Cells expressing GFP and TLR9-GFP were first used to determine the oligomeric status of TLR9. Fluorescence lifetime imaging (FLIM) was performed to show the distribution of GFP and TLR9-GFP (Supporting information (SI), Figure S1). Unlike GFP, which was found to be distributed throughout the cells, the expression pattern of TLR9-GFP was found to be distributed in specific intracellular compartments (SI, Figure S1a). Past studies have shown that TLR9 is localized within the endoplasmic reticulum (ER) and endosome [25]. Since fluorescence lifetime is sensitive to the physico-chemical environment such as the membrane potential, pH and osmotic conditions [26], [27], lifetime analysis can be used to assess biomolecular interactions and localization of targets in different microenvironments. Fluorescence lifetime imaging (FLIM) is a powerful method for separating species based on differences in the exponential decay rate of fluorescence, independent of intensity. In our experiments, the average lifetime of GFP (2.3 ns±0.02 ns) and TLR9-GFP (2.9 ns±0.03 ns) was determined by fitting the time correlated single photon counting (TCSPC) data with Eq.1 (single component, i = 1). The change in lifetime could suggest that TLR9 is predominantly distributed in the endosomal compartments whose microenvironment comprising of cargos, pH, structure among others is different from that of the cytosol.

**Figure 1:**
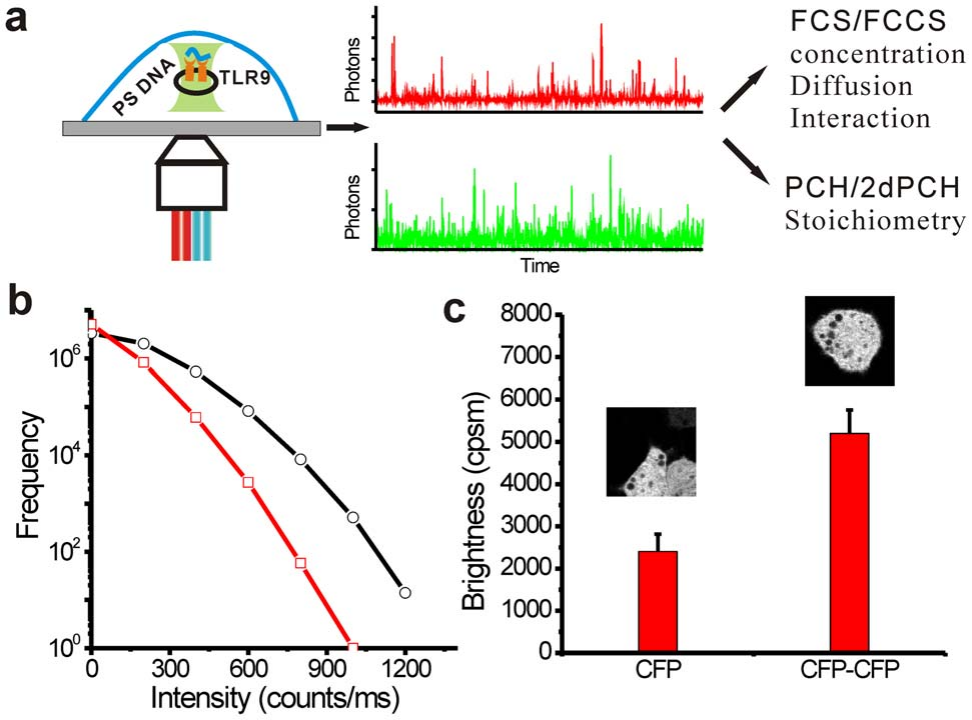
Single molecule fluorescence tools to probe TLR9 interaction in live cells. (**a**) The fluorescence fluctuation data was analyzed using the auto/cross-correlation by FCS/FCCS or PCH/2dPCH to estimate the diffusion dynamics, number of molecules, and stoichiometry. (**b**) Photon counting histogram (PCH) analysis of CFP (open red squares) and CFP-CFP (open black circles) constructs. (**c**) Calculated brightness of CFP and CFP-CFP dimer from PCH analysis. Error bars are standard deviation (n = 10). Insets are the images of HEK 293 cells transiently transfected with CFP and CFP-CFP dimer.

**Figure 2:**
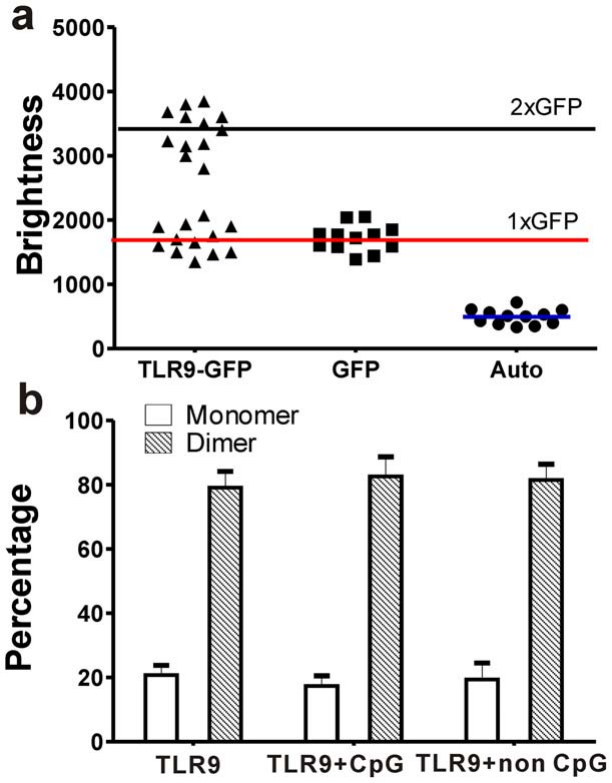
PCH analysis of TLR9-GFP dimers in live cells. (**a**) Brightness values of autofluorescence (Auto), GFP and TLR9-GFP obtained under identical experimental conditions by PCH analysis (n = 12). Blue and red line represent mean brightness counts of autofluorescence and GFP monomer brightness. Black line denotes the predicted brightness of GFP dimers. (**b**) Percentage of monomer and dimers in TLR9-GFP alone, TLR9 incubated with CpG DNA and TLR9 incubated with non-CpG DNA, quantified by PCH data. Error bars are standard deviation (n = 12).

To demonstrate the stoichiometry, HEK 293 cells were first constructed to transiently express CFP and CFP-CFP constructs (Insets, Figure 1c). PCH data (Figure 1b) of CFP and CFP-CFP expressions could be fitted well with single component (i = 1, χ^2^ = 1.32) using a very low excitation power (8 µw) to minimize photobleaching. Our results show that the brightness of CFP-CFP (2430 cpsm±375 cpsm) dimers was roughly twice that of CFP (5210 cpsm±636 cpsm), as expected, providing the basis for stoichiometry and quantification. Average brightness of GFP alone as determined by the single component (i = 1) fitting of GFP transfected cells (Figure 2a) was 1689 cpsm±321 cpsm. In contrast, PCH analysis of TLR9-GFP could be fitted (χ^2^ = 1.1) with two components (I = 2) instead of one component (i = 1, χ^2^ = 5.3). While the brightness of one segment of the population was similar to that of GFP monomers, the brightness of the other segment of the population was close to twice this value suggesting that TLR9 could homodimerize before binding to a CpG ligand. The formation of higher order oligomers of TLR9 was ruled out because the maximum brightness value observed from the TLR9-GFP was less than thrice that of monomers. Our results are consistent with the previous *in vitro* studies using co-immunoprecipitation and FRET experiments [14]. Furthermore, by global fitting of PCH data we determine that 79.1%±5.1% of the TLR9 forms dimers and the rest exist as monomers and the fraction of TLR9 dimers did not change (p = 0.8) upon addition of CpG (82.6%±6.1%) or non-CpG DNA (82.6%±4.3%) (Figure 2b). Considering the fact that TLR9 activation was restricted to phosphorothioate (PS) CpG-motif DNA [12], [13], [14], our findings suggest that its activation is not associated with CpG DNA induced formation of dimer or higher order oligomers, and additional prerequisites might be necessary for its activation.

### TLR9 binding to CpG and non CpG DNA with different affinity

**Figure 3:**
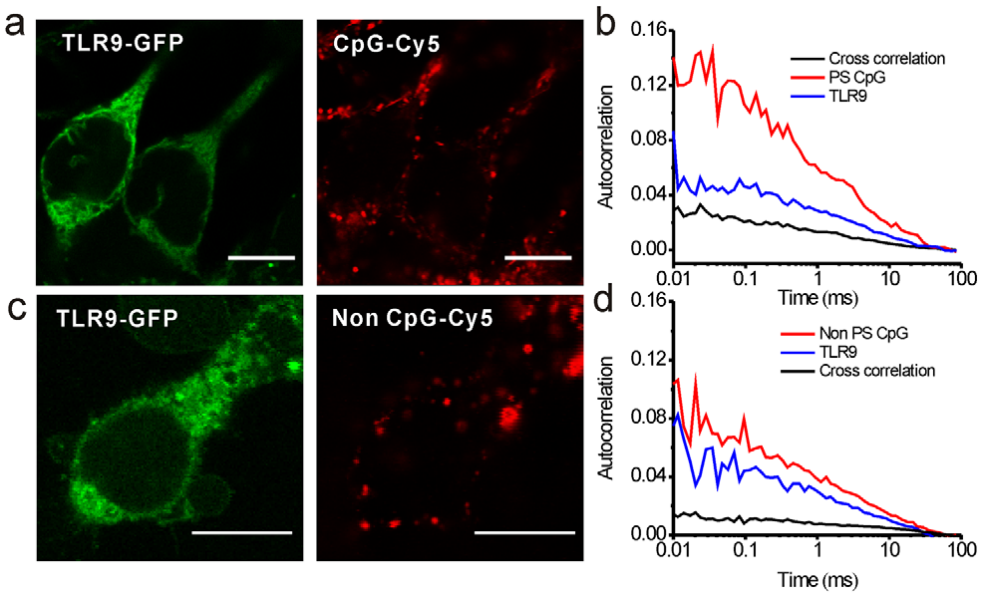
Cross-correlation analysis of TLR9 with CpG and non CpG DNA. Confocal fluorescence image of (**a**) TLR9-GFP (left) with CpG DNA (right) and (**c**) TLR9-GFP (left) with non CpG DNA (right). Scale bar: 10 µm. Autocorrelation (Blue and red) and cross-correlation (black) curves from FCCS measurement confirms the *in vivo* binding between TLR9 and CpG (**b**) or non CpG DNA (**d**). Curves represent an average of 30 different measurements across 10–30 different cells.

To investigate whether binding affinity plays an important role in TLR9 activation, we employed FCCS to evaluate the dissociation constant of TLR9 using different DNA sequences, *in vivo*. Confocal fluorescence images (Figure 3a,c) show that both CpG and non CpG DNA colocalize with TLR9-GFP, indicating a possible interaction at these regions and to provide a potential point of focus for the assessment of stoichiometry and binding kinetics. As noted earlier, with FCCS it is possible to evaluate the diffusion of two species in a focal volume by monitoring fluorescence fluctuation intensity (Figure 1a), where the amplitude of the cross-correlation provides the respective concentration of the interacting species in the complex. From the amplitude (Figure 3b,d) of the FCS and FCCS measurements, the concentration and diffusion time of TLR9, CpG or non CpG DNA and the respective bound complexes was calculated. Positive cross-correlation was obtained for both CpG and non CpG DNA bound to TLR9 and their respective dissociation constants (K_d_) were calculated in living cells using the equation, [TLR9][DNA]/[TLR9-DNA], where [TLR9] and [DNA] denote the respective concentrations of TLR9 dimers and nucleic acids. [TLR9-DNA] represents the concentration of the bound complexes. Using single molecule experiments we provide a quantitative estimate of their interactions in single cells. Calculations show that the binding affinity of TLR9 with CpG DNA (K_d_ = 62 nmol/L±9 nmol/L) was higher (p<0.01) than that of TLR9 with non CpG DNA (K_d_ = 153 nmol/L±26 nmol/L) from our live single cell experiments.

### Mobility of TLR9-Ligand complex by FCS

Since different binding affinities were noted for CpG and non CpG DNA upon binding to TLR9, we predicted that CpG-TLR9 and non CpG-TLR9 bound complex might have different intracellular mobilities. To examine the diffusion dynamics of TLR9, the diffusion time of GFP was compared with TLR9-GFP using FCS. As expected, the diffusion time of TLR9-GFP was 1.78 ms, which is greater than that of cytosolic GFP (diffusion time was 0.47 ms) at the 95% confidence limit (p<0.05). In addition, show that cytosolic GFP could be best fitted with a 3D diffusion model while the TLR9-GFP could be best fitted by an anomalous model used to describe the diffusion of particles hindered by obstacles or subject to an external force (SI, Figure S2). The anomalous diffusion exponent ‘α’ was found to be 0.35 for TLR9 which decreased in proportion to an increase in concentration of obstacles posed by intracellular constituents [28]. This value was higher than the observed value (α = 0.75) for protein [28] or lipid granules [29] diffusing in the cytoplasm, possibly due to the predominant distribution of TLR9 in the endosomes/lysosomes.

**Figure 4:**
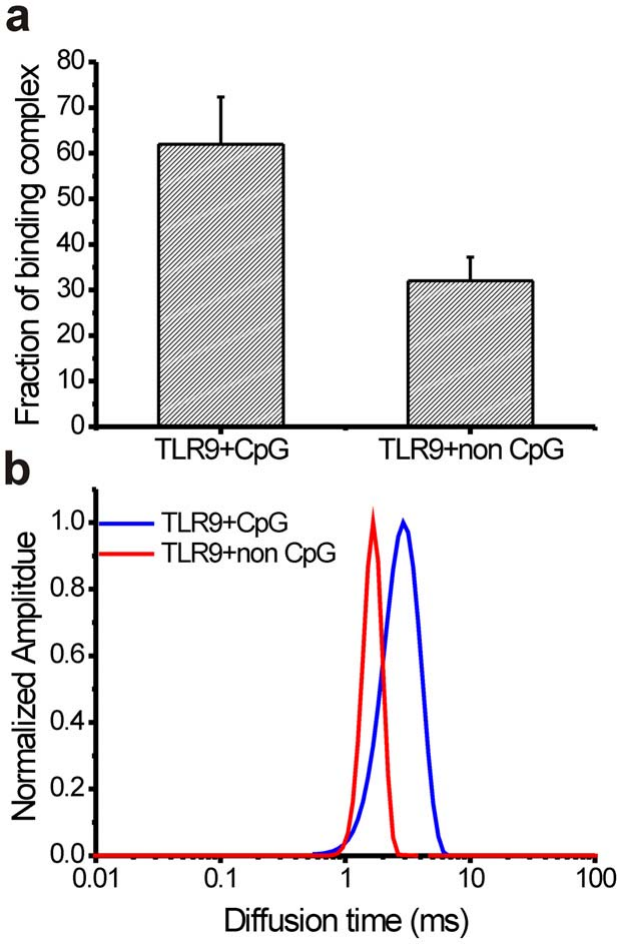
TLR9-CpG and TLR9-non-CpG has different mobility. (**a**) Fraction of the binding complex estimated from FCCS cross-correlation measurements, as a percentage of species in lower concentration of the two binding partners. Data is expressed as the mean with standard error (n = 15). (**b**) MEMFCS analysis of FCCS data shows the distribution of diffusion time in the binding complex in live HEK 293 cells.

From FCCS measurements, the concentration of the interacting species in the complex was calculated to provide an estimate of the fraction of CpG-TLR9 or non CpG-TLR9 complexes formed relative to the lower concentration of the partner (Eq. 9). Our results show that the population consisted of over 62%±11.2% of CpG DNA-TLR9 constructs compared to 32%±5.7% of non CpG DNA-TLR9 (Figure 4a) complexes in live cells (p<0.05). The diffusion characteristics of the bound complex were then calculated from FCS data using the maximum entropy method (MEMFCS)[30], which attempts to minimize the normalized chi-square value χ^2^ while maximizing the entropy ‘S’ in the calculations. Unlike conventional methods where fitting components need to be assigned an initial value, MEMFCS does not need an initial value. Further, fitting a heterogeneous population of diffusers is possible because this analysis yields a diffusion time distribution that represents a heterogeneous population. From MEMFCS we show that the CpG-TLR9 complex (peak position at 2.9 ms) has a longer diffusion time compared to non CpG-TLR9 (peak position at 1.6 ms) at the 95% level (p<0.05) (Figure 4b), indicating a possible increase in its size due to the recruitment of MyD88 and subsequent signaling complex comprising of TRAF6 (tumour-necrosis factor (TNF)-receptor-associated factor 6), BTK (Bruton's tyrosine kinase), IRAK4 (interleukin-1-receptor (IL-1R)-associated kinase 4) and IRAK1to initiate signaling [31], [32].

### Stoichiometry of the TLR9 DNA interaction in living cells

**Figure 5:**
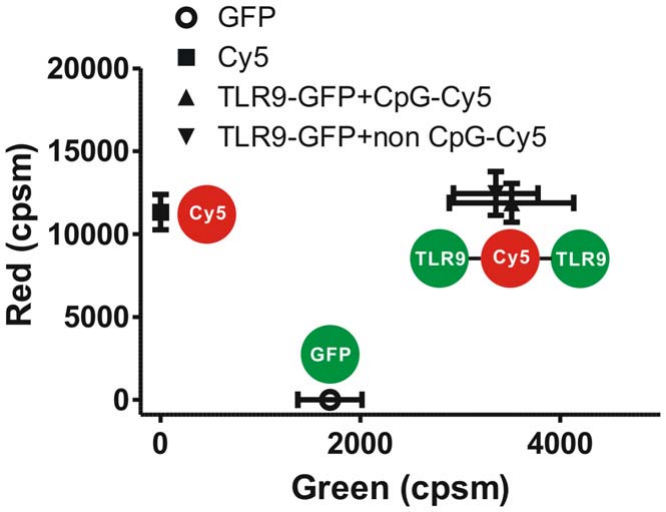
Stoichiometry of TLR9-ligand complex *in vivo*. Binding stoichiometry of TLR9-GFP with CpG-Cy5 and non CpG-Cy5 calculated from the two-dimensional PCH analysis of fluorescence fluctuation data. Data is expressed as the mean with standard deviation (n = 10).

While FCCS could provide information on concentration and diffusion time of bound molecules, two-dimensional PCH (2dPCH), an extension of regular PCH (ie. 1dPCH) developed in this work [22], is uniquely suited to provide stoichiometry information of the complex, based on its brightness information using photon counts from the two detector channels. Furthermore, since the instrument setup of 2dPCH is identical to FCCS, the fluorescence fluctuation raw data could be used for both FCCS and 2dPCH analysis. Since each detection channel records its own color of light, the 2dPCH distinguishes fluorescent species not only based on the differences in their brightness, but also according to their color for stoichiometry analysis [33], [34]. The 2dPCH histograms of CpG-TLR9 and non CpG-TLR9 complexes could be fitted well with a single component (i = 1, χ^2^ = 1.43) using the respective brightness values of the diffusing particles recorded in the green and red channels. The data shows that both the CpG-TLR9 and non CpG-TLR9 complexes have an average brightness (CpG-TLR9: 11886 cpsm±1169 cpsm, non CpG-TLR9: 12452 cpsm±1320 cpsm) comparable to that of Cy5 monomers in red channel and an average brightness similar to GFP-GFP dimers in the green channel (Figure 5), suggesting a 2∶1 (TLR-GFP:DNA-Cy5) binding stoichiometry for both CpG-TLR9 and non CpG-TLR9 complexes in live cells.

## Discussion

Traditional biochemical assay such as co-immunoprecipitation or pull down experiments are valuable tools for studying protein-protein or protein-DNA interactions. However, these approaches require the disruption and cell lysis to extract the protein of interest. Single molecule fluorescence techniques demonstarted have the ability to provide quantitative estimates of biomolecular interactions without disrupting the partnering molecules at high spatial and temporal resolution. Consequently, the work presented here successfully combines FCCS and PCH and FLIM to assess the intracellular localization, binding affinity, mobility, and composition of TLR9 and its ligands by estimating the diffusion time, number of molecules, fluorescence lifetime, brightness and stoichiometry of interaction (Figure 1a) in live single cells.

We first use PCH analysis to investigate TLR9 oligomerization before and after its interaction with DNA. By characterizing the amplitude of fluorescence fluctuations, PCH provides two estimates, the number of particles (N) and molecular brightness (ε). Molecular brightness (ε), defined as the average number of photons per molecule per second, is an inherent property of the fluorescence molecule that is not related to concentration and could provide a “brightness signature” to separate different species [21]. Therefore, one would expect the dimer constructs to approximately measure twice the brightness values of monomers. From our experiments, we observed that TLR9 predominantly forms dimers prior to binding to a ligand. By quantitatively estimating the fraction of bound complex, we found that the percentage of dimers formed did not change irrespective of the interacting partner, either the CpG or non CpG DNA (Figure 2b). Previous *in vitro* experiments confirmed that CpG containing nucleic acids could activate the downstream signaling of TLR9 by bringing the cytoplasmic domains in close proximity while non CpG could not induce conformational changes [14]. Our results show that formation of dimers was not sufficient for the activation of TLR9 and an additional prerequisite for activation constituting a change in structural conformation might be necessary. This mechanism is different from TLR3, which is capable of binding to double stranded RNA (dsRNA), to exclusively form monomer constructs in solution but in most instances forms dimers when bound to dsRNA through a highly cooperative processes [35]. These results suggest that the activation and binding model could be different for each TLRs because their ligands are fundamentally different. Future studies on *in vivo* binding to other TLRs will be essential for understanding the biology of TLRs and their interacting partners.

Since we have demonstrated that the percentage of TLR9 predimers did not increase upon addition of CpG or non CpG DNA, the binding affinity of TLR9 to different DNA motifs might play an important role in its activation. Using fluorescence cross-correlation we report the binding affinity (K_d_) of TLR9 to CpG DNA as 62 nmol/L±9 nmol/L while that of non CpG DNA was 153 nmol/L±26 nmol/L. Our observation is expectedly different from the previous *in vitro* [14] work which reported a value in the range of 1–10 nmol/L, possibly attributable to the intracellular microenvironment and the association of TLR9 with other proteins for downstream signal activation upon binding to CpG DNA. While past studies show that TLR9 activation and signaling driven by CpG DNA requires acidification and maturation of endosomes [36], the acidic pH could serve as an optimal condition for interaction and could contribute to the difference in the *in vitro* and *in vivo* binding affinity estimates. Given the fact that the activation of TLR9 involves ligand-induced conformational changes to the TLR9 homodimers, our results suggests that TLR9 conformational change and activation was induced by CpG DNA and not by non CpG DNA because of a significant difference in the dissociation constant values obtained from our *in vivo* single molecule experiments. It is worth noting that the fusion of GPF to TLR9 will not adversely affect its interaction with the CpG nucleotides because TLR9-GFP in our study was tagged at their C termini with enhanced GFP while previous study indicates that the N-terminal site in TLR9 is responsible for CpG-DNA recognition and activation [37].

The B-class oligonucleotide (ODN) used in our study as a model ligand forms stable monomer constructs instead. Therefore, although we have established the stoichiometry of the ligand binding to TLR9 through 2dPCH, the exact stoichiometry of DNA molecules could vary with the type of TLR9 ligand. Based on our results, which demonstrate that the CpG and non-CpG PS DNA have the same stoichiometry with TLR9 but with different binding affinity, it implies that the CpG and non CpG DNA might bind to different sites of TLR9. However, the location of the binding sites can only be confirmed from crystal structures of DNA-TLR9 binding domain, which is beyond the scope of our study.

In conclusion, we demonstrate a binding model based on quantitative estimates of the binding affinity of TLR9 dimers with CpG and non CPG DNA sequences in live HEK 293 cells. A multiparameter single molecule fluorescence platform was established to study the dynamics of the TLR9-ligand complex and define their stoichiometry at single cell resolution. We expect our findings to not only help further the understanding of TLR9 interactions *in vivo* but also to provide new insights towards the design and characterization of TLR9-based therapeutics for the treatment of autoimmune diseases or cancer.

## Materials and Methods

### TLR9 ligands

The phosphorothioate (PS) backbone CpG and non CpG DNA bearing the respective sequences, 5′-TCGTCGTTTTGTCGTTTTGTCGTT-3′ and 5′-TGCTGCTTGTGCTTTTGTGCTT-3′ were commercially purchased (IDT, Coralville, IA). The DNA was labeled with single Cy5 fluorophore at the 3′ end.

### Plasmid construction

To generate the CFP-CFP tandem construct, the coding sequence of enhanced CFP was PCR-amplified using 5′-aaa aga tct ccA TGG TGA GCA AGG GCG AGG-3′ and 5′-cgc tcg aga CTT GTA CAG CTC GTC CAT GCC G-3′ oligonucleotides, digested with *Bgl*II and *Xho*I, and ligated into the corresponding sites of a CFP-containing pHA-CMV vector (a kind gift from Dr. Hu, Purdue University). A GGGGSGGGGSGGGGS linker was used to separate the two CFP sequences. The fabricated construct was verified by DNA sequencing.

TLR9 (GenBank^TM^ accession number AF259262) tagged at their C termini with enhanced green fluorescent protein (EGFP) was obtained from Dr. David M. Segal (National Cancer Institute, Bethesda, MD) [38]. pEGFP-N1 was purchased from Clontech (Mountainview, CA).

### Cell culture

HEK 293 cells were grown in RPMI-1640 (ATCC) media with 10% fetal bovine serum and maintained at 37°C with 5% CO_2_. For living-cell experiments, cells were seeded onto sterilized No. l coverslip (VWR International, Batavia, IL) and placed in a 6-well plate. TLR9-GFP and EGFP pDNA was transfected using 1 µL Lipofectamine 2000 (Invitrogen, Carlsbad, CA), according to manufacturer's specifications. The amount of DNA used per well was 500 ng of EGFR-GFP.

### Single-molecule fluorescence instrument

All the single molecule fluorescence experiments were performed with a time-resolved confocal fluorescence scope fitted with picosecond lasers (465 nm and 636 nm at 20 MHz) and optics for single molecule fluorescence spectroscopy and lifetime imaging (Microtime 200, Picoquant, Berlin, Germany). Details of the instrumentation are provided elsewhere [17], [20]. The effective confocal volume (V_eff_  =  π^3/2^ω^2^z) calculated for the 465 nm and 636 nm excitation from the autocorrelation fitting functions for Rhodamine 123 (300 um^2^/s) and Atto 655 (390 um^2^/s) dyes (Invitrogen Molecular Probes Eugene, OR) were 0.36 fL and 1 fL, respectively.

### Fluorescence lifetime imaging (FLIM)

Fluorescence lifetime imaging was measured using the time correlated single photon counting (TCSPC) module in the time-tagged time-resolved (TTTR) mode. Fluorescence lifetime was obtained by fitting the TCSPC histograms with the multi-exponential model. 
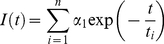
(1)


Where τ_i_ denote the lifetime with amplitudes α_i_ corresponding to component i. The values of τ_i_ and α_i_ were obtained through nonlinear least-squares fitting with the SymphoTime software (PicoQuant GmbH Berlin, Germany) and the goodness of fit was determined by χ^2^ value.

### FCS and FCCS

Fluorescence fluctuation, δI(t) around the average fluorescence <I> were recorded in real time and the normalized autocorrelation was calculated from:
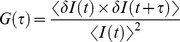
(2)δI(t) = I(t)-<I(t)>. The autocorrelation curve of fluorophore diffusing in solution was fitted to a 3D diffusion model using one or two components with the SymphoTime software and Origin Lab using the equation:



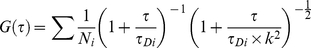
(3)


Where, N*_i_* and τ*_Di_* denote the number of fluorescent molecules in the detection volume and diffusion time of component i, respectively. The parameter ‘κ’ and the lateral diffusion coefficient ‘D’ can be obtained from:
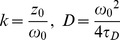
(4)


Here, κ is defined as the ratio of the axial beam size z and radius ω of the laser, and τ_D_ denote the diffusion time of the fluorophore.

Autocorrelation of TLR9-GFP in live cells was calculated using an anomalous diffusion model to fit the data as observed in the cytoplasm [28].



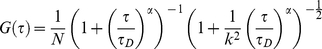
(5)


Where α represents the degree of anomalous behaviour.

For dual color cross-correlation measurement, the 465 nm and 636 nm excitation were used to respectively excite the green and red fluorophores and the cross-correlation function was calculated from,



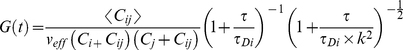
(6)


Here <C_i_>, <C_j_>, and <C_ij_> denote the concentration of species i, j, and ij (depicting the bound species), respectively, and V_eff_ is the effective detection volume for the dual color experiment. Using these considerations, the diffusion time and the effective detection volume for cross-correlation analysis can be estimated from,
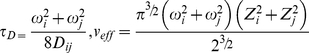
(7)


Where D_ij_ is the diffusion coefficient of the bound molecule and its concentration can be obtained from the cross-correlation analysis as,



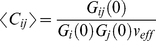
(8)


In Eq 6, G_ij_(0) is the cross-correlation amplitude at time τ = 0 and G_i_(0) and G_j_(0) are the respective autocorrelation amplitudes of species i and j at time τ = 0.

The fraction of bound complex was calculated as a percentage of the lower concentration species (C_i_ or C_j_) according to the following equation: 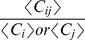
(9)


### MEMFCS

MEMFCS (Maximum Entropy Method analysis of Fluorescence Correlation Spectroscopy data) [30] was employed to validate the cross-correlation diffusion time distribution. The analysis is based on minimizing the χ^2^ value and maximizing the entropy S to obtain an optimal fit when species with different diffusion times are involved. G(τ) in Eq 3, can be rewritten to obtain a continuous distribution of diffusion times as:
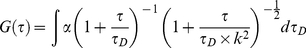
(10)


S is defined as, 
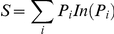
, where 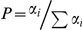.

### PCH and 2dPCH

For one-dimensional PCH analysis, data was arranged as a histogram comprising of the number of photon events per unit time with a bin size of 50 µs. From the histogram, molecular brightness and concentration of molecules were extracted [39]. The autofluorescence from live cells was taken into consideration as the background during analysis. A one (i = 1) or two component model (i = 2) was used to fit the data depending upon the χ^2^ value using the PCH and 2dPCH software [34]. The values depicting the brightness of GFP monomer and GFP dimer were fixed and the data was fitted globally to two components to determine the percentage of TLR9 monomers and dimers.

For 2dPCH analysis, the data was arranged in the form of two-dimensional histogram of counts as a function of frequency with a bin size of 50 µs, optimized for this experiment. The binding data of TLR9 and DNA was fitted with a single component and an F-test was used for parameter optimization when fitting for more than one component.

## Supporting Information

Figure S1 TLR9-GFP and GFP has different fluorescence lifetime. (**a**) Distribution pattern of GFP and TLR9-GFP by confocal fluorescence lifetime imaging. (**b**) GFP and TLR9-GFP lifetime distribution histogram corresponding to the two images in (**a**). Lifetime pseudo color bar: 0 to 5 ns. Scale bar: 10 µm.
(TIF)

Figure S2 FCS evaluation of GFP and TLR9-GFP. Autocorrelation curves of cytosolic GFP (red square) and TLR9-GFP (blue square) and the best fit curves (black). Cytosolic GFP was fitted with a 3D diffusion model (Eq 3.) while TLR9-GFP was fitted with the anomalous model (Eq 5).
(TIF)
