# Supplementary figures and images for: Single Molecule In Vivo Analysis of Toll-Like Receptor 9 and CpG DNA Interaction
Source: PLoS One. 2011 Apr 4;6(4):e17991. doi: 10.1371/journal.pone.0017991 (PMC3070698; doi:10.1371/journal.pone.0017991)

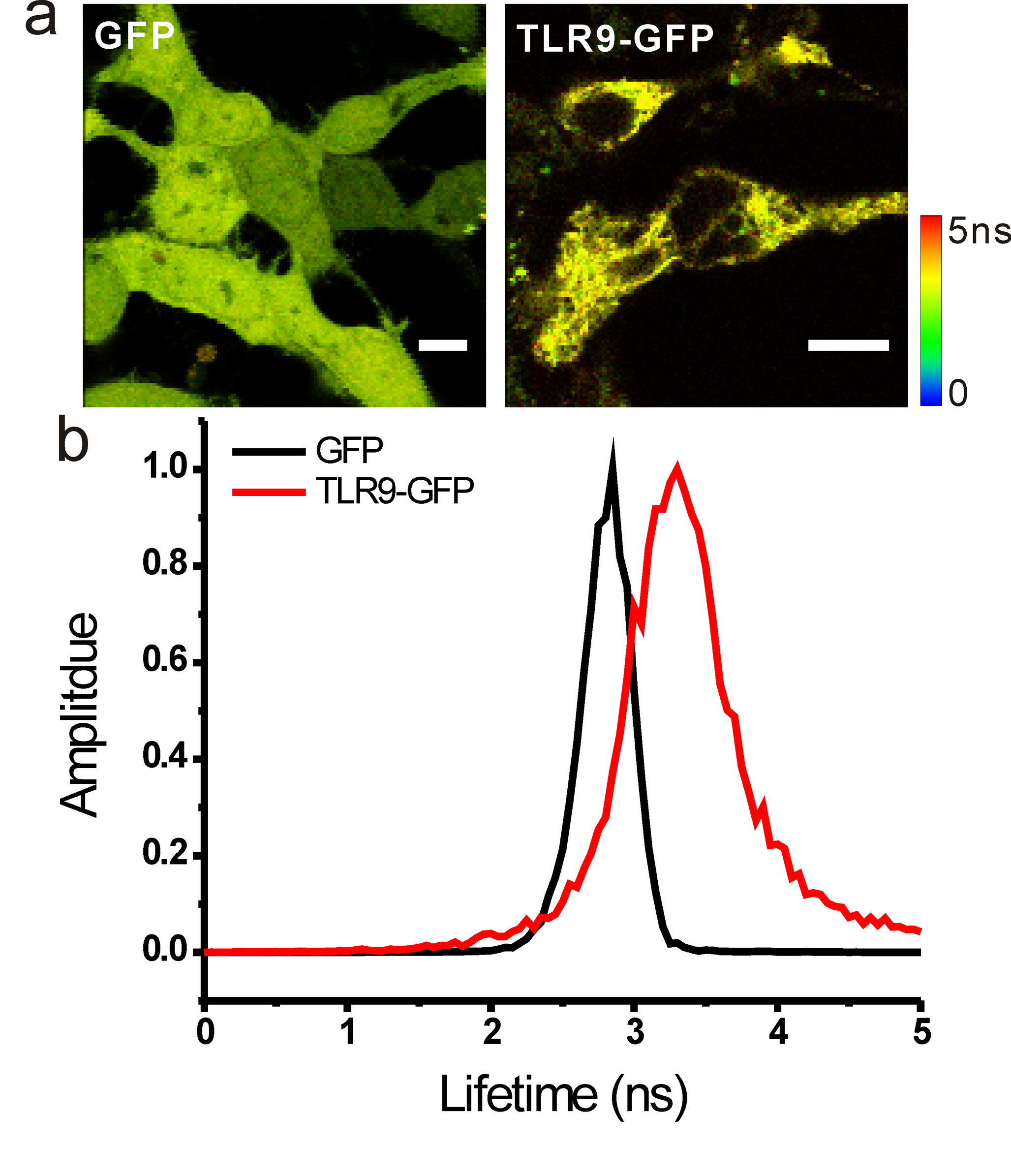

Supplement: Figure S1 — TLR9-GFP and GFP has different fluorescence lifetime. (a) Distribution pattern of GFP and TLR9-GFP by confocal fluorescence lifetime imaging. (b) GFP and TLR9-GFP lifetime distribution histogram corresponding to the two images in (a). Lifetime pseudo color bar: 0 to 5 ns. Scale bar: 10 µm. (TIF) [file pone.0017991.s001.tif]

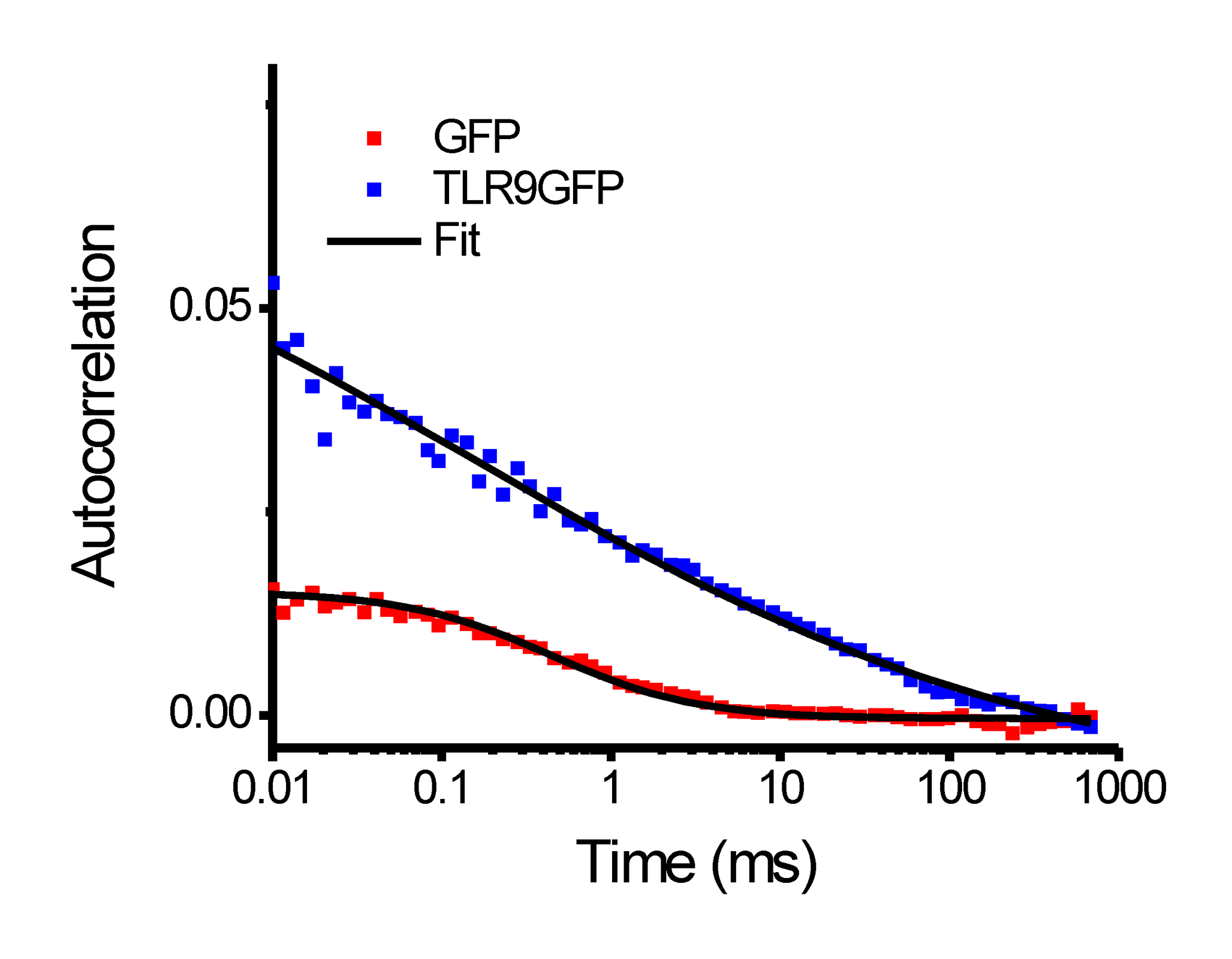

Supplement: Figure S2 — FCS evaluation of GFP and TLR9-GFP. Autocorrelation curves of cytosolic GFP (red square) and TLR9-GFP (blue square) and the best fit curves (black). Cytosolic GFP was fitted with a 3D diffusion model (Eq 3.) while TLR9-GFP was fitted with the anomalous model (Eq 5). (TIF) [file pone.0017991.s002.tif]
